# Delaying the first grapevine fungicide application reduces exposure on operators by half

**DOI:** 10.1038/s41598-020-62954-4

**Published:** 2020-04-14

**Authors:** Mathilde Chen, François Brun, Marc Raynal, David Makowski

**Affiliations:** 1 ACTA - les instituts techniques agricoles, 149 rue de Bercy, Paris cedex 12, 75595 France; 2 Université Paris-Saclay, AgroParisTech, INRAE, UMR Agronomie, 78850 Thiverval-Grignon, France; 3 Inserm U1153, CRESS, Epidemiology of Ageing and Neurodegenerative diseases, Université de Paris, Paris, France; 4 INRAE, UMR AGIR, 31326 Castanet Tolosan cedex, France; 5 IFV, Bordeaux Nouvelle Aquitaine, UMT SEVEN, 71 Avenue E Bourlaux, 33882 Villenave d’Ornon cedex, France; 6 CIRED, 45bis Avenue de la Belle Gabrielle, 94130 Nogent-sur-Marne, France

**Keywords:** Plant breeding, Agroecology, Risk factors

## Abstract

Downy mildew is a severe disease of grapevines treated by repeated fungicide applications during the growing season. The impact of these treatments on human health is currently under scrutiny. Fungicide application long before disease onset is not thought to be greatly beneficial for grape production, but the first fungicide treatment is applied at least six weeks before disease onset in more than 50% of the vineyards in the Bordeaux region, a major French vine-growing area. We estimate that applying one fungicide every two weeks at disease onset would reduce fungicide applications against downy mildew by 56% (95%IC = [51.0%, 61.3%]), on average, relative to current levels. This decrease is slightly greater than the level of exposure reduction resulting from the random suppression of one out of every two fungicide treatments (i.e. 50%). The reduction is lower when treatments are sprayed weekly but still reaches at least 12.4% (95%IC = [4.3%, 20.8%]) in this case. We show that this and other strategies reducing the number of treatments would decrease operator exposure to pesticides as effectively as the use of various types of personal protective equipments in the Bordeaux region. The implementation of this strategy would significantly decrease fungicide use, health risks, and adverse environmental impacts of vineyards.

## Introduction

Downy mildew, caused by the oomycete *Plasmopara viticola*, is a major disease of grapevine worldwide. If left untreated, grape downy mildew (GDM) attacks can cause yield losses^1,2^ and reduce grape quality^3^. This disease is currently controlled mostly by intensive pesticide use, particularly in Bordeaux, a major vine-growing area in France^4^. In this region, many vine growers start applying fungicides early in spring, and spraying is then repeated regularly throughout the growing season, about two or three times per month, depending on their action period. This strategy keeps damages levels low but entails a large number of fungicidal treatments (7.9 treatments per vineyard and year in average in France^5^), with potential environmental implications, high production costs^6^ and risks to human health, particularly for vine growers^7–11^.

Several approaches for reducing pesticide exposure have been proposed. The use of personal protective equipment (PPE) is often promoted as an efficient mean of reducing the exposure of operators (the individuals applying the treatments^12^) to pesticides. However, it was pointed out that PPE was not systematically worn during pesticide applications^13^. Indeed, wearing PPE appears to be uncomfortable in certain climatic conditions and in certain positions^14,15^. Another limitation of this approach is its inability to reduce exposure of residents living close to vineyards. Concern about the exposure of residents to vineyards where pesticides are applied has increased sharply in recent years^16,17^.

Operator exposure could also be decreased by reducing fungicide use, as recommended by the Ecophyto II + Plan, initiated in 2018 in France following the European Directive on the sustainable use of pesticides in the EU Member States^18^. This national plan aims to reduce the use of plant protection products by 25% in 2020 and 50% in 2025^19^.

Delaying the first application for GDM treatment could potentially reduce the total number of fungicide treatments over the growing season. Mailly *et al*.^20^ showed, in a nationwide survey, that the total number of fungicide applications in the vineyard was smaller for growers who began spraying later in the season (i.e. after May 15^th^). GDM can be efficiently controlled by fungicides beginning at the time of infection^6^ or a few days before the symptoms onset predicted by forecasting models^21^. However, most vine growers in the Bordeaux region currently start spraying fungicides before mid-May^20^, several weeks before first GDM symptoms onset^22^. Postponing the first fungicide treatment to the date of symptom onset could, thus, potentially reduce the number of fungicide treatments and the associated exposure to pesticides. However, the degree to which fungicide use and operator exposure can be reduced by this approach has never been quantified.

In this study, we estimated the dates of the first GDM treatment in the Bordeaux vineyards from different sources of information. Based on our estimations, we calculated the potential decrease in fungicide use relative to current practices in this region that could be achieved by delaying the first spray until symptom onset. This assessment was based on different treatment frequency assumptions, ranging from one fungicide spray every two weeks to one spray per week. These decreases were converted into decreases in operator exposure and compared with the decrease in exposure associated with the use of various types of PPE, computed with a model predicting the exposure of agricultural operator^23^. We found that postponing the first fungicide application to disease onset would decrease operator exposure to pesticides by the same order of magnitude as the use of PPE.

## Results

### Large gap between first fungicide spray and the date of disease onset

**Figure 1:**
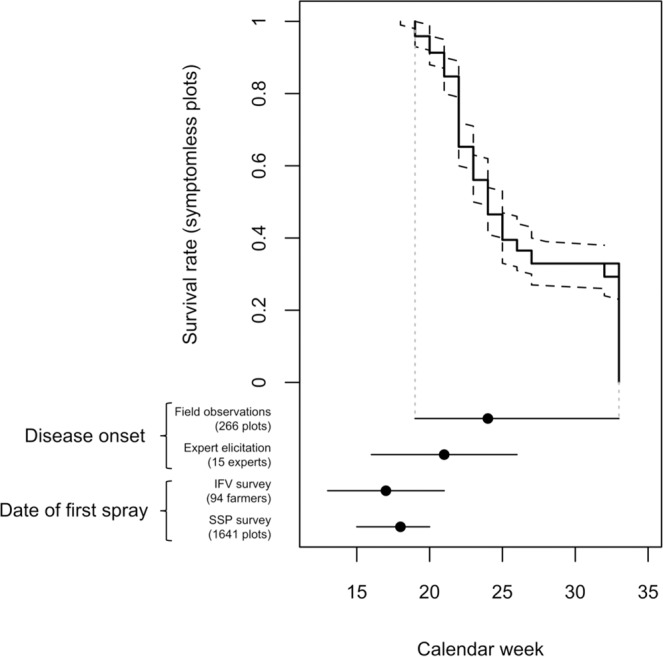
Proportion of symptomless plots based on observations collected in 266 sites-years (decreasing survival curve in plain lines with 95% confidence intervals in dashed lines). Horizontal bars represent the 2.5% and 97.5% percentiles and points correspond to median dates computed from different sources of information, from top to bottom: dates of onset of the first GDM symptoms based on observed plots and on expert elicitations, first GDM fungicide spraying dates according to two surveys, IFV and SSP surveys.

The date of first GDM treatment ranges from calendar week (cw) 12 (mid-late March) to cw 28 (early-mid July), according to a survey conducted by the French extension service for vines and wines (*Institut Français de la Vigne et du Vin*, referred to hereafter as IFV) in the Bordeaux region in 2018 (Fig. 1). According to this source of information, about 95% of the questioned vine growers in the Bordeaux region applied their first GDM treatment between cw 12 (mid-late March) and cw 21 (mid-late May) (Fig. 1). These results were confirmed by a three-year survey conducted by the French Ministry of Agriculture’s Statistics and Prospective Service (SSP), which found that about 95% of the Bordeaux vine growers questioned applied their first GDM treatment between cw 15 (mid April) and cw 20 (mid May) (Fig. 1).

Although both farm surveys showed that 90% of vineyards received their first treatment against GDM before cw 19 (early-mid May), our statistical survival analysis of the on-field GDM disease observations revealed that almost none of the 266 untreated site-years, i.e. the combination of monitored vineyard site and year, displayed any symptoms of GDM before cw 19. Here, the first symptoms of the disease appeared in less than 10% (95%CI = [3.7%, 12.8%]) of the plots by cw 20 (mid-late May), and in 50% (95%CI = [46.8%, 59.7%]) of the plots by cw 24 (mid-June), i.e. six to eight weeks after the median date of first fungicide application reported in the farm surveys (Fig. 1). By cw 32 (mid August), the proportion of plots with GDM symptoms remained below 75% (95%CI = [61.0%, 73.5%]). We found a relationship between the date of symptom onset and spring rainfall. The median date of GDM onset was delayed by about three weeks, i.e. 50% (95%CI = [36.6%, 64.0%]) of the monitored plots displayed GDM symptoms by cw 27 (early July) if the spring was dry (mean daily rainfall < 1.51 mm/day in March-June, like in 2011). The first symptoms appeared in 50% (95%CI = [52.1%, 67.4%]) of the plots by cw 24 (mid-June) in wet springs (mean daily rainfall > 5.45 mm/day in March-June, like in 2013). By cw 33 (mid-late August), GDM onset was reported for less than 60% of the plots (95%CI = [37.4%, 70.0%]) and for more than 70% (95%CI = [68.4%, 85.9%]) of the plots in dry and wet springs, respectively.

Regional experts (vine growers’ advisers) tend to anticipate the dates of first GDM symptom appearance relative to field observations. The median date of symptom onset estimated by elicitation of 15 regional experts is cw 21 (late May), three to five weeks after the median date of first fungicide treatment reported in agricultural surveys but about two weeks before the median date of symptom onset in the untreated fields monitored. The probability of GDM onset was 90% (95%IC = [82.0%, 96.0%]) by cw 24 (mid-June), based on regional experts elicitation, when the proportion of infected plots remains inferior to 55%, according to our survival analysis (Cox model).

### Delaying the date of the first spray could halve fungicide use

**Figure 2:**
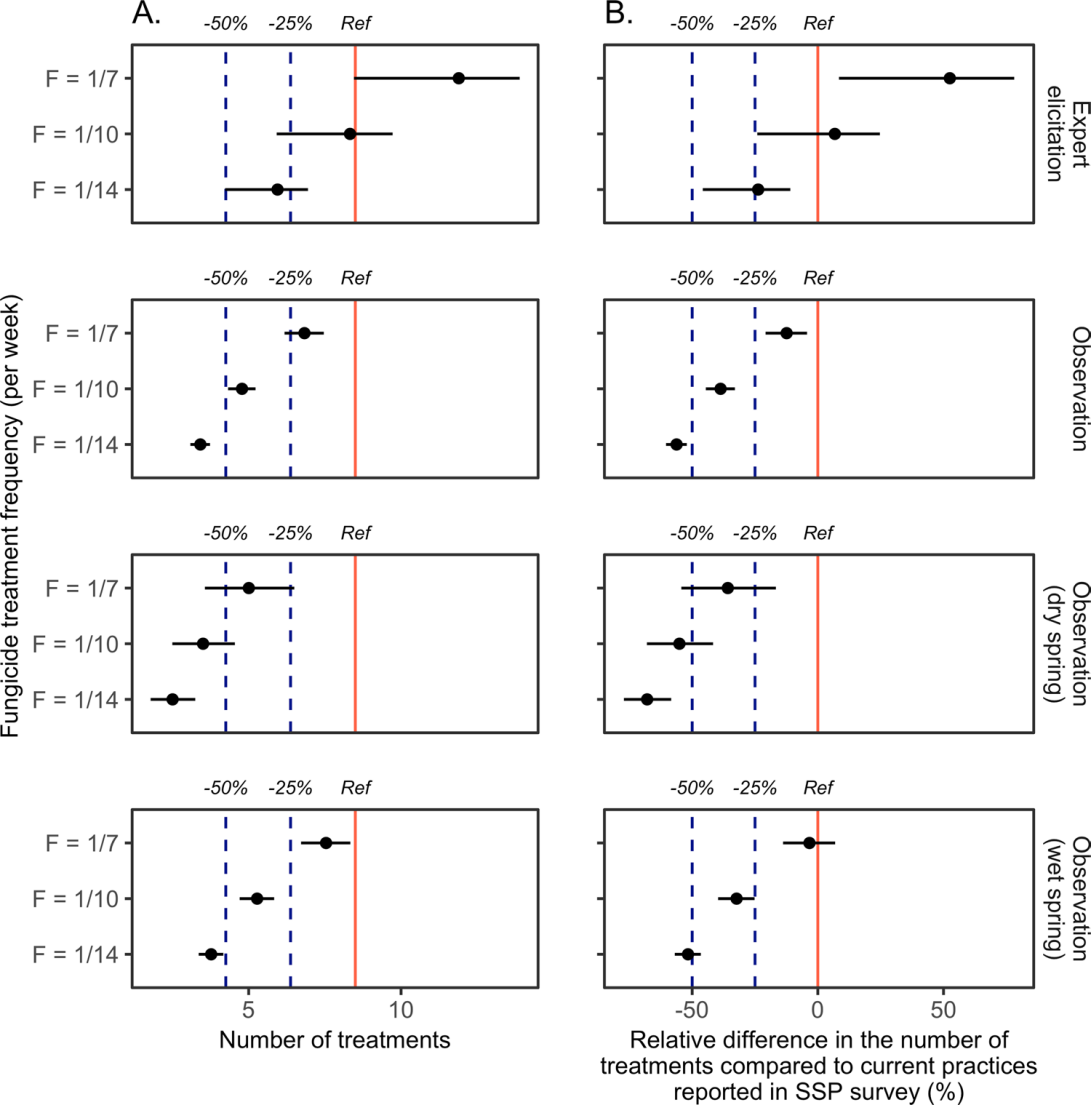
(**A**) Number of anti-GDM fungicide sprays for different strategies to trigger the first fungicide application (based on expert elicitation or on observed dates of disease onset) and different treatment frequencies (F = 1/7, i.e., one treatment per week, F = 1/10, i.e., one treatment every 10 days, F = 1/14, i.e., one treatment every two weeks). Vertical red plain line represents the number of fungicide applications corresponding to current farmers’ practices according to the SSP survey (“Ref” line). Reduction levels of −25% and −50% compared to current practices are represented by vertical blue dashed lines, respectively (“−25%” and “−50%” lines). (**B**) Same results expressed as relative differences in the number of fungicide applications (%) for different strategies to trigger the first fungicide application, based on expert elicitation and observed dates of disease onset, respectively, combined with different treatment frequencies compared to current farmers’ practices according to the SSP survey. Horizontal lines represent 95% confidence intervals in both panels. Results based on IFV survey are presented in Supplementary Fig. SF1.

Assuming that fungicides are usually applied every two weeks after the first application^24^, the median number of fungicide applications would be 8.5 and 7.8 based on the dates of the first fungicide spray reported in the IFV and SSP farm surveys, respectively. This result is consistent with the number of fungicide applications reported by the SSP for the 2010 and 2013 growing seasons in the Bordeaux region^5,25^. Assuming a treatment frequency of one treatment every two weeks, delaying the first fungicide spray until the date of disease onset, derived from field observations, would result in 3.4 fungicide applications per year, on average for all years (Fig. 2A and Supplementary Fig. SF1A). It corresponds to a 59.9% (95%IC = [55.0%, 64.5%]) and 56.0% (95%IC = [51.0%, 61.3%]) decrease relative to the numbers of treatments derived from the IFV and SSP surveys, respectively (Fig. 2B and Supplementary Fig. SF1B).

However, the actual decrease in the number of fungicide treatments depends on climate conditions in the spring. In dry springs, the decrease may be as high as 70.4% (95%IC = [61.3%, 79.1%]) and 67.6% (95%IC = [57.8%, 77.3%]) (2.5 treatments) relative to the numbers of treatments computed from the IFV and SSP surveys, respectively. In a wet spring, the decrease would be lower, at 55.6% (95%IC = [49.8%, 61.2%]) and 51.4% (95%IC = [45.3%, 57.8%]) (3.8 treatments) relative to the numbers of treatments estimated from the IFV and SSP surveys, respectively.

The use of a higher treatment frequency of one application per week combined with a delay of the first application to the disease onset results in a smaller decrease in the number of treatments equal to 19.6% (95% CI = [12.2%, 27.3%]) and 12.4% (95% CI = [4.3%, 20.8%]) compared to current practices of farmers reported in the IFV and SSP surveys, respectively.

Experts tend to predict earlier dates of first symptom appearance than those actually observed in the field (Fig. 1). Thus, smaller decreases would be achieved if the first fungicide is sprayed on the date of disease onset estimated from expert knowledge. Assuming a frequency of one treatment every two weeks, the number of treatments would be about 29.4% (95%IC = [26.3%, 35.4%]) and 22.7% (95%IC = [19.7%, 29.6%]) lower than that derived from the IFV and SSP surveys, respectively. On the other hand, with a frequency of one treatment every week, the number of treatments would be 40.0% (95%IC = [0.0%, 63.5%]) and 52.5% (95%IC = [8.4%, 78.1%]) higher than that derived from the IFV and SSP surveys, respectively.

### Means of decreasing operator exposure to fungicides

**Figure 3:**
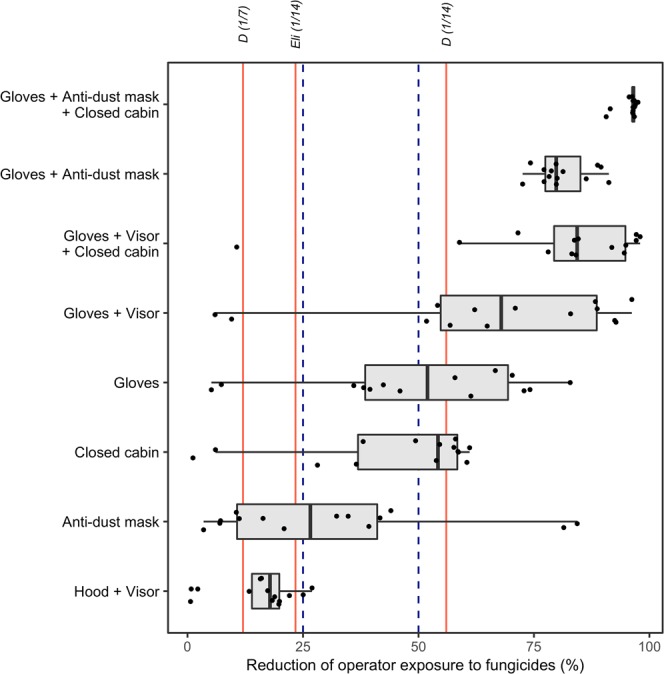
Levels of reduction of operator exposure to 13 fungicide molecules for various operator protection scenarios. Each point represents the reduction of exposure to one molecule resulting from the use of personal protective equipments. Red vertical lines indicate the median decrease in exposure achieved by different first treatment postponing strategies, specified above each line (“D (1/7)” = first application delayed until disease onset estimated by survival analysis + treatment applied every week; “D (1/14)” = same delay + treatment applied every two weeks; “Eli (1/14)” = first application delayed until disease onset estimated by probabilistic expert elicitation + treatment applied every two weeks). Blue dashed vertical lines represent 25% and 50% exposure reduction, respectively.

Decreasing the number of GDM fungicide sprays by delaying the first fungicide treatment until disease onset results in a lower level of operator exposure to fungicides. We compared the decrease in exposure due to this strategy with that resulting from the use of personal protective equipment (PPE). Operator exposure to the main active compounds of GDM fungicides was calculated with the exposure model of Großkopf *et al*.^23^, for different combinations of PPEs (Fig. 3, Supplementary Table S1 and Supplementary Fig. SF2).

For several active substances, a reduction in the number of fungicide treatments is more protective than wearing certain PPEs when mixing, loading and applying fungicides. Among the reduction treatment strategies considered here, delaying the first fungicide application until disease onset induces the greatest decrease in exposure. However, other strategies also result in significant reductions in exposure. With a treatment frequency of one spray every two weeks, delaying the first application of the fungicide is more protective than wearing a face shield with a hood or an anti-dust mask for most substances. Considering the data for all years together, the use of a closed tractor cabin did not reduce operator exposure more than reducing the number of fungicide treatments by delaying the first fungicide spray until disease onset for 57% of the substances considered. In dry springs, this was the case for 100% of the substances considered, whereas, in wet springs, it was the case for 43%. Considering the data for all years together, wearing gloves was less protective than postponing the first GDM treatment for 50% of the studied substances. For 36% of the substances, wearing gloves was still more protective than reducing the number of treatments, even if there was little rainfall in spring.

**Figure 4:**
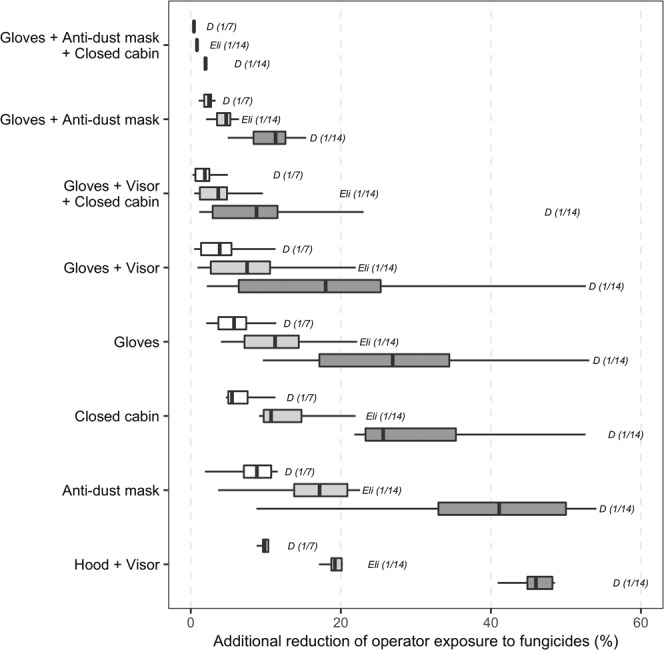
Additional reduction of operator exposure resulting from delayed first anti-GDM treatment combined with various operator protection scenarios, according to different GDM control strategies ("D (1/7)" = first application delayed until disease onset estimated by survival analysis + treatment applied every week; "D (1/14)" = same delay + treatment applied every two weeks; "Eli (1/14)" = first application delayed until disease onset estimated by probabilistic expert elicitation + treatment applied every two weeks).

**Figure 5:**
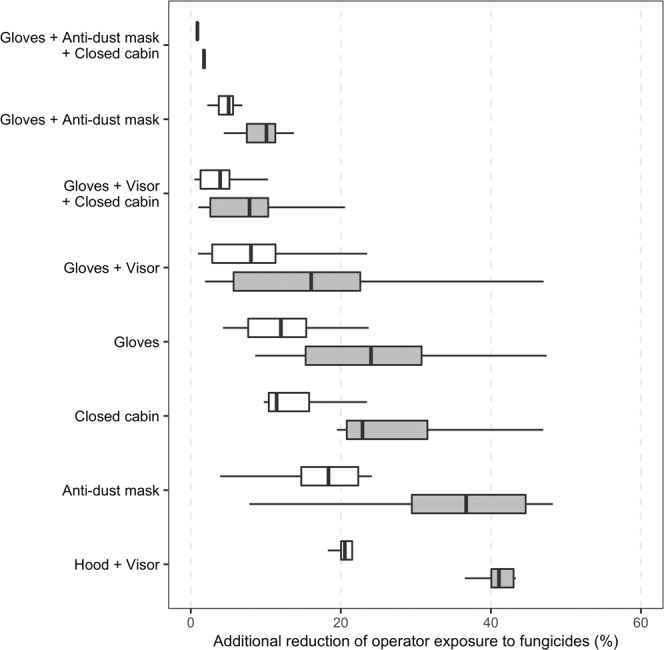
Additional reduction of operator exposure resulting from different operator protection scenarios combined with 25 and 50% fungicide application reduction scenarios. White and grey boxplots correspond to an exposure reduction of 25% and 50%, respectively.

The decrease in operator exposure was enhanced by using several PPEs together during pesticide mixing, loading and application. On average, the use of both gloves and a face shield was more protective than delaying the first fungicide treatment until disease onset for 71% of the active substances. The largest decrease in exposure was achieved through the combined use of gloves and anti-dust mask. Furthermore, this combination of PPEs was also more effective at reducing exposure than delaying first fungicide application until disease onset. However, even for combinations of several PPEs, operator exposure was further reduced by delaying the first fungicide treatment until disease onset (Fig. 4 and Supplementary Fig. SF3). This delay systematically gave substantial exposure reduction for every studied PPE combinations. The strongest decrease in operator exposure was observed when postponing first application was combined with the use of a hood and visor (from 16.6 to 67.1%, depending on the substance) and the smallest was achieved with a combination of delayed spraying and the use of gloves, anti-dust mask and a closed cabin (from 0.6 to 6.3%). The levels of exposure reduction resulting from the random suppression of one out of every two fungicide treatments (i.e. 50%) were slightly lower (Fig. 5).

The decrease in exposure was much lower when the date of first symptoms appearance was estimated from expert knowledge. This finding is consistent with the anticipation of symptom appearance by the experts; the decrease in the number of fungicide treatments and the associated decrease in exposure were smaller when the first fungicide application took place on the dates of symptom appearance estimated by the experts than when they took place on the dates derived from field observations.

Levels of exposure reduction were smaller when the treatment frequency was increased from one treatment every two weeks to one treatment every week. Indeed, an increase in the frequency of treatment partially offsets the effect of the postponement of the first treatment on operators’ exposure. However, combined with PPE, triggering first fungicide application at disease onset was still able to reduce operators’ exposure by 2.4 to 11.9% (Fig. 4, see results for “D (1/7)”).

## Discussion

In Bordeaux vineyards, GDM is mainly controlled by fungicide sprays. In 2013, a mean of 10.1 fungicide applications were used to control GDM in this region, accounting for 44.3% of all pesticide applications in Bordeaux vineyards that year^24^. Our results show that postponing the fungicide spray against GDM until disease onset could reduce the number of GDM fungicide treatments by 56.0%, on average, relative to current agricultural practices in Bordeaux area. This reduction level is slightly greater than the level resulting from the random suppression of one out of every two fungicide treatments (i.e. 50%). Delaying first GDM fungicide application at first symptoms appearance date could eliminate a mean of 5.7 fungicide treatments, corresponding to 25.0% of all pesticide applications applied on average in this year. Thus, this approach would substantially contribute to the Ecophyto II + plan, implemented in 2018 to reduce fungicide use by 25% in 2020 and by 50% in 2025 in France^19^. Some vine growers may be tempted to increase the frequency of treatments if there is a delay in the date of the first anti-GDM spray. It is therefore possible that the frequency of treatment may increase to one treatment per week, but it is unlikely that the frequency of treatment will exceed this level, as the minimum persistence time for anti-GDM fungicides is at least seven days^26^. An increase in the treatment frequency partially offsets the benefit of postponing first treatment at GDM onset on fungicide use reduction. However, we showed that, even with a frequency of one treatment per week, triggering first fungicide application at disease onset was still able to reduce fungicide applications compared to current practices.

Our estimations are consistent with the conclusion of Mailly *et al*.^20^, who showed, based on the results of a national survey over two growing seasons (2006 and 2010), that fungicide applications were halved if vine growers did not start applying fungicides until after May 15th. Based on an epidemiological model, Caffi *et al*.^6^ showed that the number of fungicide treatments could be reduced by 33 to 86%, with a median reduction of 54%, relative to standard practices in northern Italy, if fungicide spraying was delayed until an infection was predicted. In the same study, the authors showed that this strategy protected grape crops effectively against the disease. Menesatti *et al*.^21^ reported a similar decrease in fungicide applications for delaying the first fungicide treatment against GDM until predicted date of disease onset, associated with an efficient level of control of the pathogen.

We used the AOEM model^23^, recommended by European Food Safety Authority (EFSA)^12^, to assess operator exposure in various PPE scenarios. AOEM is an exposure model based on data collected from recent, representative and reproducible studies. Other exposure models, such as the UK POEM and German models, exist^27^ but these models are based on old data, and are therefore not representative of current agricultural practices, particularly for the use of different types of PPE by operators^12^. The AOEM model calculates dermal exposure of the body assuming that the operator is wearing normal work clothes, defined as at least one layer of work clothing completely covering the body, arms and legs^23^, and a full-body PPE overall is not considered. This is a limitation of the model as the combination of gown and working overall tested by Thouvenin *et al*.^28^ may further reduce operators’ exposure.

Our results show that a reduction in the number of fungicide treatments contributes to a substantial reduction in operators’ exposure to fungicides. The highest exposure reduction was observed when the date of the first fungicide treatment was delayed until disease onset. We show that this strategy induced similar exposure reduction than certain PPE. Our study is the first comparing the decrease in exposure yielded by various types of PPE combined with reduced number of GDM treatments. Furthermore, previous studies have focused only on a small number of molecules, such as dithiocarbamates, folpet^29^, arsenic^30^ and spinosad^28^, whereas we estimated the decrease in operator exposure to 13 molecules commonly used to control GDM in the Bordeaux region. We found that delaying the first anti-GDM treatment reduced operator exposure to these active molecules to a greater extent than wearing some forms of PPE (gloves, hood with a visor and anti-dust mask) during mixing, loading and application. These results are consistent with those of Baldi *et al*.^31^, who showed that wearing PPE, such as gloves, did not significantly decrease operator exposure to dithiocarbamates (e.g. mancozeb) or folpet. Our results also show that the use of closed tractor cabin during pesticide application was not more protective than postponing the first GDM treatment for more than half of the considered substances. However, the highest levels of exposure reduction were obtained in scenarios combining PPEs and a delay of first GDM treatment.

Operator exposure to pesticides used in viticulture is thought to affect the development of several diseases in the vine growers population, including cancers^7,16^, Parkinson’s disease^9,32^, and neurological problems^10,33^, particularly in Bordeaux vineyards^8^. Mancozeb, one of the most widely used anti-GDM molecule in Bordeaux vineyards according to SSP survey results (Table 1 in Material and methods section), is suspected to increase the risks of the operator developing leukemia, melanoma and Parkinson’s disease^34^. The use of PPE is generally recommended to protect operators against the adverse effects of pesticides for human health. However, PPE is frequently used incorrectly or not at all^13,35^, resulting in incomplete protection of operators^30,31,36^. Moreover, several studies have highlighted the impact of pesticide exposure on residents in the surrounding area^32,37^, who cannot be protected by PPE. In this context, delaying the time of first anti-GDM spraying until disease onset could efficiently decrease the risks of residents exposure to fungicides. However, as GDM often occurs earlier in damp spring conditions than in dry springs, the decreases in the number of treatments and associated exposure probably vary from year to year.

**Table 1:** Main active substances, their associated fungicide products and maximum authorized sprayed quantity of each active substance used in each product used in the Bordeaux region to control GDM.

Active substance name	Fungicide name	Maximum authorized quantity of active substance during one treatment (in kg per hectare)
Ametoctradin	ENERVIN	0.3
Benalaxyl-M	SIDECAR	0.1
Copper compounds	HELIOCUIVRE	1.94
Cyazofamid	MILDICUT	0.112
Cymoxanil	VALIANT FLASH	0.12
Dimethomorph	FORUM TOP	0.225
Disodium phosphonate	MILDICUT	1.12
Fluopicolide	PROFILER	0.133
Folpet	EPYLOG FLASH	1.5
Fosetyl	EPYLOG FLASH	1.5
Mancozeb	SIDECAR	1.62
Mandipropamid	PERGADO F PEPITE	0.125
Metalaxyl-M	RIDGOLD F	0.097
Metiram	ENERVIN	1.1
Potassium phosphonates	LBG-01F34	2.92

The maximum permitted doses of pesticide were considered to compute the amounts of active substance used by an operator.

The practicality of delaying the first fungicide treatment against GDM should be assessed in close collaboration with farmers and agricultural extension services. Several approaches have already been proposed to reduce fungicide applications by adapting vineyard phytosanitary treatments to local risks. For example, Delière *et al*.^38^ relied on several climate-based indicators, field observations and local expert knowledge to trigger fungicide treatments against GDM. In their approach, the first treatment against GDM was delayed if no symptoms were observed prior the BBCH-stage 20 of grape (i.e. more than nine unfolded leaves^39^) but a fungicide treatment was systematically scheduled at flowering, even in the absence of symptom.

Delaying the first treatment against GDM is sometimes perceived as risky by some vine growers who consider that GDM epidemics are mainly driven by secondary infections after disease onset. Asexual contamination is often assumed to play a major role in disease spreading over time and space considering that the epidemic starts from a restricted number of primary infections, followed by massive clonal reproduction. However, this conventional view has recently been seriously challenged by Gobbin *et al*.^40^ who have observed a continuous influx of new GDM genotypes into epidemics occurring in Europe and, more particularly, in the Bordeaux region. They also showed that the majority of GDM genotypes lacked the capacity to generate secondary lesions^41^. These results suggest that the primary inoculum plays a much more important role in GDM epidemics than is usually considered, while the secondary inoculum has a generally low and variable degree of success in generating secondary infections.

Several experiments have been conducted to evaluate the consequences of triggering a fungicide spray at the beginning of GDM. In their study, Menesatti *et al*.^21^ evaluated three strategies for the control of GDM in a two-year field experiment: no treatment, standard control strategy consistent with current farmer practices, and first fungicide spray at the predicted dates of disease onset. The dates of disease onset are rarely measured directly in practice and, when they are, they are often censored because field observers do not usually visit vineyards every day^22^. For these reasons, dates of disease onset usually need to be estimated using expert knowledge, statistical analyses, or predictive models. Menesatti *et al*.^21^ used a statistical model to forecast the date of GDM onset (with a prediction accuracy of 81%) and to trigger first fungicide applications at predicted dates. Their results showed that triggering the first fungicide application at predicted disease onset effectively controlled GDM and reduced the number of fungicide applications by almost half. When the first fungicide spray was applied at forecasted disease onset, the levels of disease incidence and severity on leaves and bunches were significantly reduced compared to untreated controls and were similar to those obtained with a standard control strategy.

These findings are supported by the results of Jermini *et al*.^3^ and Pereira *et al*.^42^. Jermini *et al*.^3^ showed that triggering the first treatment when the first symptoms appeared was effective in controlling the GDM epidemic and preserving grape production in Switzerland. These results support the assumption that postponing the first GDM treatment at disease onset contributes to effectively control the disease and to reduce the number of fungicide applications compared to conventional protection strategies. Between 2013 and 2015, Pereira *et al*.^42^ conducted a field experiment where GDM severity on bunches was assessed weekly in Brazilian vineyards. They compared two treatment strategies against GDM: a conventional approach based on systematic treatments and a strategy in which treatments were started only after the onset of the disease and until fruit ripening. The authors showed that postponing the date of first treatment after disease onset did not increase GDM severity on bunches compared to a conventional treatment strategy. Although the experiments of Pereira *et al*.^42^ were conducted using a tolerant grape variety, the pattern of spread of the epidemic seemed similar to that observed on susceptible grape varieties. As the number of experimental studies is limited, it would be useful to confirm these results by carrying out new experiments covering various agricultural and environmental conditions.

The approach presented here could benefit from various tools, such as weekly alert bulletins regularly published in major vine-growing areas. These bulletins report forecasts of disease occurrence based on annual field surveys^43^. Climate and/or phenological indicators^6,44^ could also be used to estimate the date of GDM onset and to follow GDM outbreak evolution. Model forecasts can be used to predict disease onset date and timing of first fungicide application^21^. It may be possible to estimate the date of disease onset more precisely in the future, by using on-farm measurements collected by sensors on drones^45^ and systems for the detection of inoculum in the vineyard^46^. The economic benefits of reducing the number of fungicide treatments remain to be assessed. The unreliable efficacy of biocontrol agents^47^ and tolerant vine varieties^47,48^ and the lower wine quality associated with resistant cultivars^49^ mean that systematic use of fungicide treatments remains the reference method of controlling GDM. However, in a context of growing concerns about the impact of pesticides on the environment and human health, regulations on chemical pesticide use may become more restrictive in the future.

## Materials and methods

### Dates of first fungicide treatment

#### SSP survey

Pesticide use on vines in the Bordeaux region was surveyed by SSP during the 2010, 2013 and 2016 growing seasons, on 606, 576 and 459 vineyards (*n* = 1641), respectively. Information about product names, dates and rates of pesticide application were collected in each vineyard and the date of first fungicide application to control downy mildew was determined for all vineyard*year combinations.

#### Survey conducted by the French Vine and Wine Institute

In spring 2018, IFV published an online questionnaire to collect data from vine growers on the timing of the first anti-GDM treatment applied. Vine growers in the Bordeaux region (*n* = 94) were asked to report the frequency of treatments against GDM for each 10-day interval between March 1 and July 31. The protocol of the farm survey was approved by the IFV to ensure that the data source is valid and that the data is used correctly and ethically. The responses received were used to plot histograms and to determine likely dates of first treatment. A continuous probability distribution was fitted to each histogram with the *fitdist()* function of version 1.4.0 of the SHELF package^50^ of R^51^ (version 3.3). The distribution yielding the best quality of fit was selected for estimation of the median date of first GDM spray for each vine grower.

### Dates of GDM onset

#### Survival analysis

Dates of GDM onset were estimated by analyzing epidemiological data collected from untreated plots in vineyards of the Bordeaux region. GDM incidence data were collected from 266 vineyards in the Bordeaux region from 2010 to 2017 by the technical staff of the IFV. In each vineyard, at least one untreated row of vine stocks was monitored for the detection of GDM symptoms. The number of vine stocks with GDM symptoms was determined by visual inspection. Visual observations stopped when the proportions of infected vine stocks and bunches were close to 100%. In total, 1 to 19 GDM incidence data were collected in each vineyard. Cox survival models^52^ were fitted to estimate the dates at which the epidemiological threshold of 1% of vines infected was reached in the sample of untreated vines surveyed. The results obtained with the fitted Cox model were used to derive three distributions of dates of GDM onset: a common distribution for all years, and two separate distributions for years characterized by dry spring (rainfall in March-May < 1.51 mm/day) and years with a wet spring (rainfall in March-May > 5.45 mm/day), respectively. 95% confidence intervals were computed from 1000 bootstrap samples. A full description of the statistical procedure is presented elsewhere^22^.

#### Expert probabilistic elicitation

Fifteen experts (technical advisors to vine growers) were individually asked to estimate the dates on which a threshold of 1% of vines displaying disease (i.e., GDM onset) would be reached in untreated plots in the Bordeaux region in 2017–2018. The experts were asked to assess the probability of the epidemiological threshold being reached for each 10-day interval between March 1 and July 31. Each participant gave a reply in the form of a histogram, according to the guidelines of the “roulette” method^53^. Several probability distributions were then automatically adjusted to fit each histogram and the distribution giving the best fit was selected. Each elicitation was carried out with MATCH Tool®^53^ and resulted histograms were analyzed using  the *fitdist()* function of version 1.4.0 of the SHELF package^50^ of R^51^ (version 3.3). This procedure was applied by each expert, each year, between April 15 and May 15, generating one distribution of dates of GDM onset per expert*year combination.

### Number of fungicide treatments

For each distribution of dates of first anti-GDM treatment or of disease onset dates, we calculated a mean number of fungicide sprays in vineyards of the Bordeaux region. Each distribution was used to compute the proportion of vineyards in which fungicide treatment begins for each week, between cw 12 (mid-late March) and cw 33 (mid August). These proportions were then multiplied by the duration of the treatment period (number of weeks) and by the treatment frequency.

Based on each probability distribution, the mean number of fungicide applications against GDM (NT) in the Bordeaux vineyards was calculated as

$$NT=\mathop{\sum }\limits_{{t}_{i}={t}_{S}}^{{t}_{F}}{\omega }_{{t}_{i}}({t}_{F}-{t}_{i})F$$

where *t*_*i*_ is the week of the first treatment (based on the distributions of dates of first GDM application and disease onset, estimated as explained above), *t*_*F*_ is the week of the last treatment, ${\omega }_{{t}_{i}}$ is the proportion of vineyards in which fungicide treatment begins on date *t*_*i*_, and *F* is the treatment frequency, in weeks. The values of ${\omega }_{{t}_{i}}$ were calculated from the probability distributions.

Computations derived from current practices in Bordeaux vineyards were made using a value of *F* based on local practices; according to national reports on fungicide use in French vineyards, vine growers apply fungicide every two weeks on average, i.e. $F=1/2$^24^. Some farmers may be tempted to increase the frequency of treatments if there is a delay in the date of the first anti-GDM spray. To estimate the impact of an increase in treatment frequency, two other values of F, F = 1 (one treatment per week) and F = 7/10 (one treatment every 10 days), were used to calculate the values of NT derived from the survival analysis and expert elicitation. It is unlikely that the frequency of treatment will exceed one per week, as the minimum persistence time for anti-GDM fungicides is at least seven days^26^.

### Assessment of operator exposure to fungicides

#### AOEM model

The AOEM model is a statistical model developed by Großkopf *et al*.^23^ for the prediction of agricultural operator exposure in treatment scenarios representative of current agricultural practices in EU member states^23^. Operator exposure (in mg per kg of body weight per day) corresponds to the exposure of a professional operator during a whole working day spent mixing, loading and applying plant protection products. The exposure due to the rinsing of the containers or vessels and the cleaning and maintenance of the equipment is also taken into account.

Inhalation, head, ‘inner’ body (i.e. exposure of the body in the absence of special PPE use but with normal work clothes), ‘total’ body (i.e. sum of inner and outer body exposure), protected hand and unprotected hand exposure are modeled separately with log-linear equations in the form of $\log (X)=\alpha \,\log (A)+\sum [{F}_{i}]$ where *X* is the exposure value (in mg per kg of body weight per day), *A* is the total amount of active substance used per operator per day and *F*_*i*_ is a set of categorical factors. The final form of the equation for each term depends on the pesticide mixing/loading and application scenarios considered.

Terms relating to dermal exposure (including the head, body and hand terms) and inhalation are used for the calculation of dermal exposure ($D{E}_{O}$) and inhalation exposure ($I{E}_{O}$) to an active substance, respectively. The assessment of $D{E}_{O}$ and $I{E}_{O}$ also takes into account absorption through the skin or by inhalation (according to the term assessed) of the active substance, the default body weight of the operator (i.e. 60 kilograms) and a risk mitigation factor corresponding to the use of PPE (if indicated). The model then estimates overall operator exposure (in mg per kg of body weight per day) as the sum of $D{E}_{O}$ and $I{E}_{O}$.

Based on new exposure datasets from 34 unpublished studies^23^, the AOEM model is representative of current agricultural practices, including PPE use, in EU member states^12^. Furthermore, the selection of these 34 studies was transparently explained, making the outputs of the model reproducible. EFSA considered this exposure model to be suitable for inclusion in its guidelines on operators’ exposure assessment and in its exposure calculator^12^. All calculations and model equations were included in the exposure calculator spreadsheet provided by EFSA^12^. This calculator is an electronic tool that uses the input provided to assess the exposure of operators, workers, residents and bystanders for each application scenario chosen^12^. The “Operator Outdoor Spray AOEM” section of this calculator (in which the AOEM model of Großkopf *et al*.^23^ is implemented) was used to assess operator exposure to the main active substances used to control GDM in the Bordeaux region according to different protection scenarios, as described below.

### Main active substances used in Bordeaux vineyards against GDM

Use frequencies of the most common fungicides applied to control GDM in the Bordeaux region were calculated from the SSP surveys. Each fungicide product was characterized in terms of the mean number of sprays in a given vineyard and the number of vineyards in which it was used during each of the growing seasons surveyed (2010, 2013 and 2016).

The composition of each fungicide (i.e. names of active substances and amount of each molecule in the fungicide) was obtained from the ANSES E-Phy database^54^. Substances and fungicide products were paired, and the number of applications of each pair in the region was calculated by multiplying the mean number of treatments with the fungicide by the number of vineyards receiving these treatments.

The total number of treatments containing each active substance was calculated by summing the number of applications of the pairs containing the substance. The 13 most frequently used substances and substance*product combinations were selected (Table 1).

### Operator protection scenarios

Operator exposure to each selected molecule was calculated for nine scenarios involving different combinations of PPE: i.e. gloves, face shield (referred to as a “visor” in the model options) worn with a hood, and anti-dust masks (FP2, P2 or similar type), during mixing, loading and application of the pesticide (Table 2). The closure of the tractor cabin door during pesticide application was simulated in three of these scenarios. A scenario with no PPE was also simulated. Each scenario was simulated for the 13 most frequently used substance*product combinations. In total, 126 simulations were performed in the “Operator Outdoor Spray AOEM” section of the EFSA calculator (Supplementary Table S1).

**Table 2:** Operator protection scenarios involving PPE use and a closed tractor cabin.

Scenario ID	Mixing and loading		Application		
	Gloves	Head and respiratory PPE	Gloves	Head and respiratory PPE	Closed cabin
1	Yes	Anti-dust mask	Yes	Anti-dust mask	Yes
2	Yes	Hood and visor	Yes	Hood and visor	Yes
3	No	None	No	None	Yes
4	Yes	Anti-dust mask	Yes	Anti-dust mask	No
5	No	Anti-dust mask	No	Anti-dust mask	No
6	Yes	Hood and visor	Yes	Hood and visor	No
7	No	Hood and visor	No	Hood and visor	No
8	Yes	None	Yes	None	No
9	No	None	No	None	No

Exposure assessments were used to calculate the decrease in exposure resulting from the use of each item of PPE individually or in combination as $RE{D}_{i}=\frac{({E}_{0}-{E}_{i})}{{E}_{0}}$, with ${E}_{0}$ operator exposure estimated for the scenario without PPE and ${E}_{i}$ operator exposure estimated for the scenario involving the PPE or combination of PPE *i*.

Decreases in exposure due to the use of combinations of PPE were combined with those resulting from delaying the first fungicide treatment until disease onset, to assess the total reduction of exposure as $RE{D}_{ij}=1\,(1-RE{D}_{i})(1-RE{D}_{j}$), where $RE{D}_{i}$ is the reduction resulting from using the *i*^*th*^ combination of PPE, $RE{D}_{j}$ is the reduction resulting from delaying the first anti-GDM treatment until the date of disease onset estimated with the *j*^*th*^ estimation method (i.e. early or late expert probabilistic elicitation, null Cox model or Cox model including weather cofactors).

## Supplementary information

Supplementary information.

## References

[1] Dubos, B. Maladies cryptogamiques de la vigne - Les champignons parasites des organes herbacés et du bois de la vigne. (2002).

[2] Gessler C, Pertot I, Perazzolli M (2011). Plasmopara viticola: a review of knowledge on downy mildew of grapevine and effective disease management. Phytopathologia Mediterranea.

[3] Jermini, M., Blaise, P. & Gessler, C. Quantitative effect of leaf damage caused by downy mildew (*Plasmopara viticola*) on growth and yield quality of grapevine ‘Merlot’ (*Vitis vinifera*). *VITIS - Journal of Grapevine Research* **49**, 77–85 (2010).

[4] Commission européenne. The use of plant protection products in the European Union, data 1992–2003. (2007).

[5] Service de la Statistique et de la Prospection. Enquête Pratique culturales en viticulture 2013 - Nombre de traitements phytosanitaires, http://agreste.agriculture.gouv.fr/IMG/pdf/dossier28_integral.pdf (2015).

[6] Caffi T, Rossi V, Bugiani R (2010). Evaluation of a warning system for controlling primary infections of grapevine downy mildew. Plant disease.

[7] Viel JF, Challier B (1995). Bladder cancer among French farmers: does exposure to pesticides in vineyards play a part?. Occupational and Environmental Medicine.

[8] Baldi I (2001). Neuropsychologic effects of long-term exposure to pesticides: results from the French Phytoner study. Environmental Health Perspectives.

[9] Baldi I (2003). Association between Parkinson’s disease and exposure to pesticides in southwestern France. Neuroepidemiology.

[10] Baldi I (2003). Neurodegenerative diseases and exposure to pesticides in the elderly. American Journal of Epidemiology.

[11] Provost D (2007). Brain tumours and exposure to pesticides: a case-control study in southwestern France. Occupational and Environmental Medicine.

[12] European Food Safety Authority (2014). Guidance on the assessment of exposure of operators, workers, residents and bystanders in risk assessment for plant protection products: Guidance on pesticides exposure assessment of operators, workers, residents and bystanders. EFSA Journal.

[13] ANSES. AVIS de l’Agence nationale de sécurité sanitaire de l’alimentation, de l’environnement et du travail relatif à l’efficacité de vêtements de protection portés par les applicateurs de produits phytopharmaceutiques - Saisine n° 2011-SA-0216. 40, https://www.anses.fr/fr/system/files/PHYTO2011sa0216.pdf (2014).

[14] Garrigou, A., Baldi, I. & Jackson, M. The use of pesticides in French viticulture: a badly controlled technology transfert. Work 19–25, 10.3233/WOR-2012-0130-19 (2012). 10.3233/WOR-2012-0130-19 22316694

[15] Grimbuhler S, Viel J-F (2018). Physiological strain in French vineyard workers wearing protective equipment to conduct re-entry tasks in humid conditions. Annals of Work Exposures and Health.

[16] Seidler A (2008). Cancer risk among residents of Rhineland-Palatinate winegrowing communities: a cancer-registry based ecological study. Journal of Occupational Medicine and Toxicology.

[17] Kab S (2017). Agricultural activities and the incidence of Parkinson’s disease in the general French population. European Journal of Epidemiology.

[18] Journal officiel de l’Union européenne. Directive 2009/128/CE du Parlement européen et du Conseil du 21 octobre 2009 instaurant un cadre d’action communautaire pour parvenir à une utilisation des pesticides compatible avec le développement durable (Texte présentant de l’intérêt pour l’EEE). 309 vol. OJ L (2009).

[19] Ministère de l’Agriculture, de l’Agroalimentaire et de la Forêt. Plan ECOPHYTO II+ (2018).

[20] Mailly F, Hossard L, Barbier J-M, Thiollet-Scholtus M, Gary C (2017). Quantifying the impact of crop protection practices on pesticide use in wine-growing systems. European Journal of Agronomy.

[21] Menesatti P (2015). Multivariate forecasting model to optimize management of grape downy mildew control. VITIS - Journal of Grapevine Research.

[22] Chen, M., Brun, F., Raynal, M. & Makowski, D. Timing of grape downy mildew onset in Bordeaux vineyards. *Phytopathology* **109**, 787–795 (2018). 10.1094/PHYTO-12-17-0412-R 30376440

[23] Großkopf C (2013). A new model for the prediction of agricultural operator exposure during professional application of plant protection products in outdoor crops. Journal für Verbraucherschutz und Lebensmittelsicherheit.

[24] Service de la Statistique et de la Prospection. Pratiques culturales en viticulture 2013 - Réduire la dose, une pratique répandue pour les traitements fongicides. 8, http://agreste.agriculture.gouv.fr/IMG/pdf/primeur343.pdf (2016).

[25] Service de la Statistique et de la Prospection. Fortes disparités de protection contre l’oïdium et le mildiou. 8, http://agreste.agriculture.gouv.fr/IMG/pdf/primeur289.pdf (2012).

[26] Chambre d’Agriculture du Tarn, Chambre d’Agriculture de Haute-Garonne, Chambre d’Agriculture du Lot & Chambre d’Agriculture du Tarn-et-Garonne. Tableau fongicides (2019). (2019).

[27] Ross J, Driver J, Lunchick C, O’Mahony C (2015). Models for estimating human exposure to pesticides. Outlooks on Pest Management.

[28] Thouvenin I, Bouneb F, Mercier T (2017). Operator dermal exposure and protection provided by personal protective equipment and working coveralls during mixing/loading, application and sprayer cleaning in vineyards. International Journal of Occupational Safety and Ergonomics.

[29] Baldi I (2012). Levels and determinants of pesticide exposure in operators involved in treatment of vineyards: results of the PESTEXPO Study. Journal of Exposure Science & Environmental Epidemiology.

[30] Grillet JP (2004). Arsenic exposure in the wine growing industry in ten French departments. Int Arch Occup Environ Health.

[31] Baldi I (2006). Pesticide contamination of workers in vineyards in France. J Expo Sci Environ Epidemiol.

[32] Kab, S., Moisan, F., Spinosi, J., Chaperon, L. & Elbaz, A. Incidence de la maladie de Parkinson chez les agriculteurs et en population générale en fonction des caractéristiques agricoles des cantons français. Bulletin épidémiologique hebdomadaire 157–167 (2018).

[33] Baldi I (2011). Neurobehavioral effects of long-term exposure to pesticides: results from the 4-year follow-up of the PHYTONER Study. Occupational and Environmental Medicine.

[34] INSERM. Pesticides - Effets Sur La Santé. (Inserm, 2013).

[35] Damalas CA, Abdollahzadeh G (2016). Farmers’ use of personal protective equipment during handling of plant protection products: Determinants of implementation. Science of The Total Environment.

[36] Lebailly P (2009). Exposure to pesticides in open-field farming in France. Ann Occup Hyg.

[37] Damalas CA, Eleftherohorinos IG (2011). Pesticide exposure, safety issues, and risk assessment indicators. International Journal of Environmental Research and Public Health.

[38] Delière L, Cartolaro P, Léger B, Naud O (2015). Field evaluation of an expertise-based formal decision system for fungicide management of grapevine downy and powdery mildews: Decision system for management of grapevine mildews. Pest Management Science.

[39] Lorenz DH (1995). Growth Stages of the Grapevine: Phenological growth stages of the grapevine (Vitis vinifera L. ssp. vinifera)—Codes and descriptions according to the extended BBCH scale†. Australian Journal of Grape and Wine Research.

[40] Gobbin D (2005). Importance of secondary inoculum of Plasmopara viticola to epidemics of grapevine downy mildew. Plant Pathology.

[41] Gobbin D, Pertot I, Gessler C (2003). Genetic structure of a Plasmopara viticola population in an isolated Italian mountain vineyard. Journal of Phytopathology.

[42] Pereira CB (2018). Temporal dynamics and management of downy mildew on the table grape ‘BRS Vitória’ in northern Paraná. Semina: Ciências Agrárias.

[43] Michel L, Brun F, Makowski D (2017). A framework based on generalised linear mixed models for analysing pest and disease surveys. Crop Protection.

[44] Kennelly MM, Gadoury DM, Wilcox WF, Magarey PA, Seem RC (2007). Primary infection, lesion productivity, and survival of sporangia in the grapevine downy mildew pathogen Plasmopara viticola. Phytopathology.

[45] Rieder, R., Pavan, W., Carré Maciel, J. M., Cunha Fernandes, J. M. & Sarroglia Pinho, M. A virtual reality system to monitor and control diseases in strawberry with drones: a project. *7th Intl. Congress on Env. Modelling and Software*. San Diego, CA, USA (2014).

[46] Thiessen LD (2016). Development of a grower-conducted inoculum detection assay for management of grape powdery mildew. Plant Pathology.

[47] Dagostin S, Schärer H-J, Pertot I, Tamm L (2011). Are there alternatives to copper for controlling grapevine downy mildew in organic viticulture?. Crop Protection.

[48] Pertot I (2017). A critical review of plant protection tools for reducing pesticide use on grapevine and new perspectives for the implementation of IPM in viticulture. Crop Protection.

[49] Espinoza, A. F., Hubert, A., Raineau, Y., Franc, C. & Giraud-Héraud, É. Resistant grape varieties and market acceptance: an evaluation based on experimental economics. OENO One **52** (2018).

[50] Oakley, J. E. SHELF: tools to support the Sheffield elicitation framework. R package version 1.4.0. (2018).

[51] R Core Team. R: A language and environment for statistical computing. The R Project for Statistical Computing, https://www.r-project.org/.

[52] Cox DR (1972). Regression models and life-tables. Journal of the Royal Statistical Society. Series B (Methodological).

[53] Morris DE, Oakley JE, Crowe JA (2014). A web-based tool for eliciting probability distributions from experts. Environmental Modelling & Software.

[54] ANSES. E-Phy - Le catalogue des produits phytopharmaceutiques et de leurs usages, des matières fertilisantes et des supports de culture autorisés en France, https://ephy.anses.fr/ (2019).

