# Supplementary material for: Delaying the first grapevine fungicide application reduces exposure on operators by half
Source: Sci Rep. 2020 Apr 14;10:6404. doi: 10.1038/s41598-020-62954-4 (PMC7156528; doi:10.1038/s41598-020-62954-4)
Supplement: Supplementary file 1 — Supplementary information. [file 41598_2020_62954_MOESM1_ESM.docx]

# Delaying the first grapevine fungicide application reduces exposure on operators by half – Supplementary information

**Authors:** Mathilde Chen^1,2,3^ *, François Brun^1,4^, Marc Raynal^5^, David Makowski^2, 6^

^1^ ACTA - les instituts techniques agricoles, 147 rue de Bercy, 75595 Paris cedex 12, France.

^2^ Université Paris-Saclay, AgroParisTech, INRAE, UMR Agronomie, 78850 Thiverval Grignon, France

^3^ Inserm U1153, CRESS, Epidemiology of Ageing and Neurodegenerative diseases, Université de Paris, Paris, France

^4^ INRAE, UMR AGIR, F-31326 Castanet Tolosan, France

^5^ IFV, Bordeaux Nouvelle Aquitaine. UMT SEVEN, 71 Avenue E Bourlaux 33882 Villenave d’Ornon Cedex

^6^ CIRED, 45bis Avenue de la Belle Gabrielle, 94130 Nogent-sur-Marne, France

*** Corresponding author**: M. Chen

**E-mail**: [mathilde.chen@acta.asso.fr](mailto:mathilde.chen@acta.asso.fr)


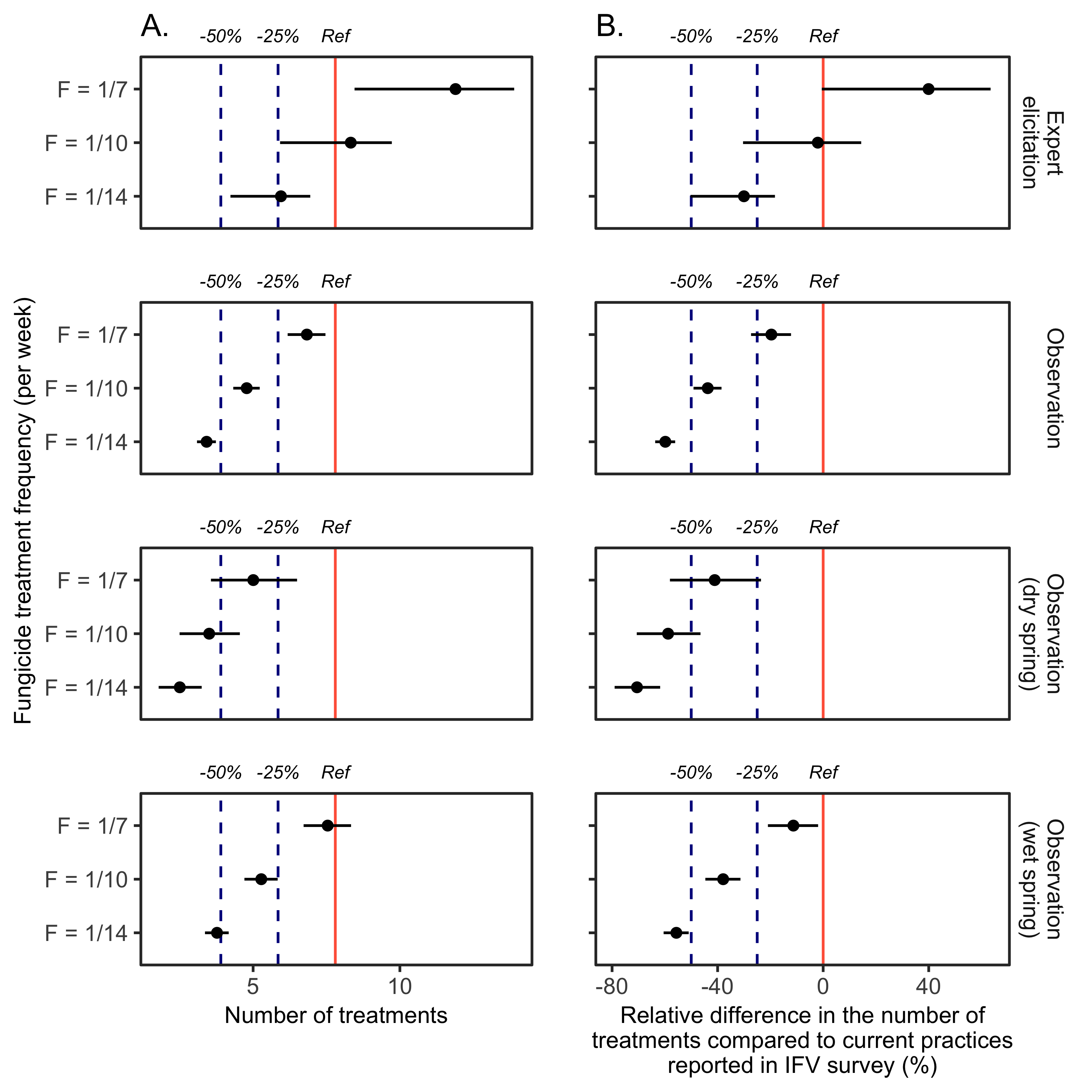


Supplementary Figure SF1: A: Number of anti-GDM fungicide sprays for different strategies to trigger the first fungicide application (based on expert elicitation or on observed dates of disease onset) and different treatment frequencies (F=1/7, i.e., one treatment per week, F=1/10, i.e. one treatment every 10 days, F=1/14, i.e., 1 treatment every two weeks). Vertical red plain line represents the number of fungicide applications corresponding to current farmers’ practices according to the IFV survey (“Ref” line). Reduction levels of -25% and -50% compared to current practices are represented by vertical blue dashed lines, respectively (“-25%” and “-50%” lines). B: Same results expressed as relative differences in the number of fungicide applications (%) for different strategies to trigger the first fungicide application, based on expert elicitation or on observed dates of disease onset, respectively, combined with different treatment frequencies and compared to current farmers’ practices according to the IFV survey. Horizontal lines represent 95% confidence intervals in both panels.


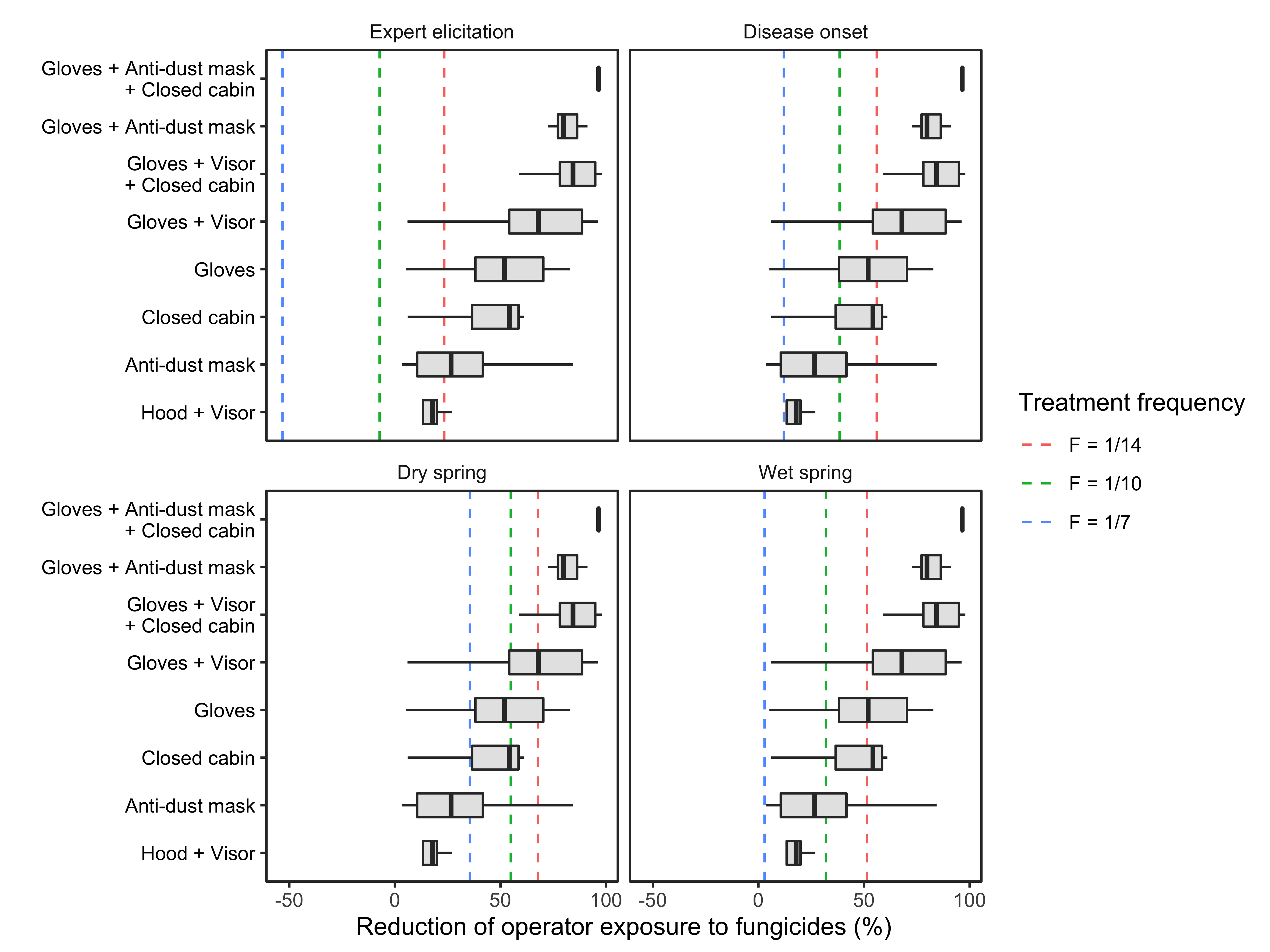


Supplementary Figure 2: Levels of reduction of operator exposure to 14 fungicide molecules for various operator protection scenarios. Vertical lines indicate the median decrease in exposure achieved by different control strategies. Each cell represents results for different date of the first application and the color of the line indicates treatment frequency. In each cell, the lower and upper hinges of the boxes correspond to the first and third quartiles (the 25th and 75th percentiles) and horizontal segment represent the range between min and max values.


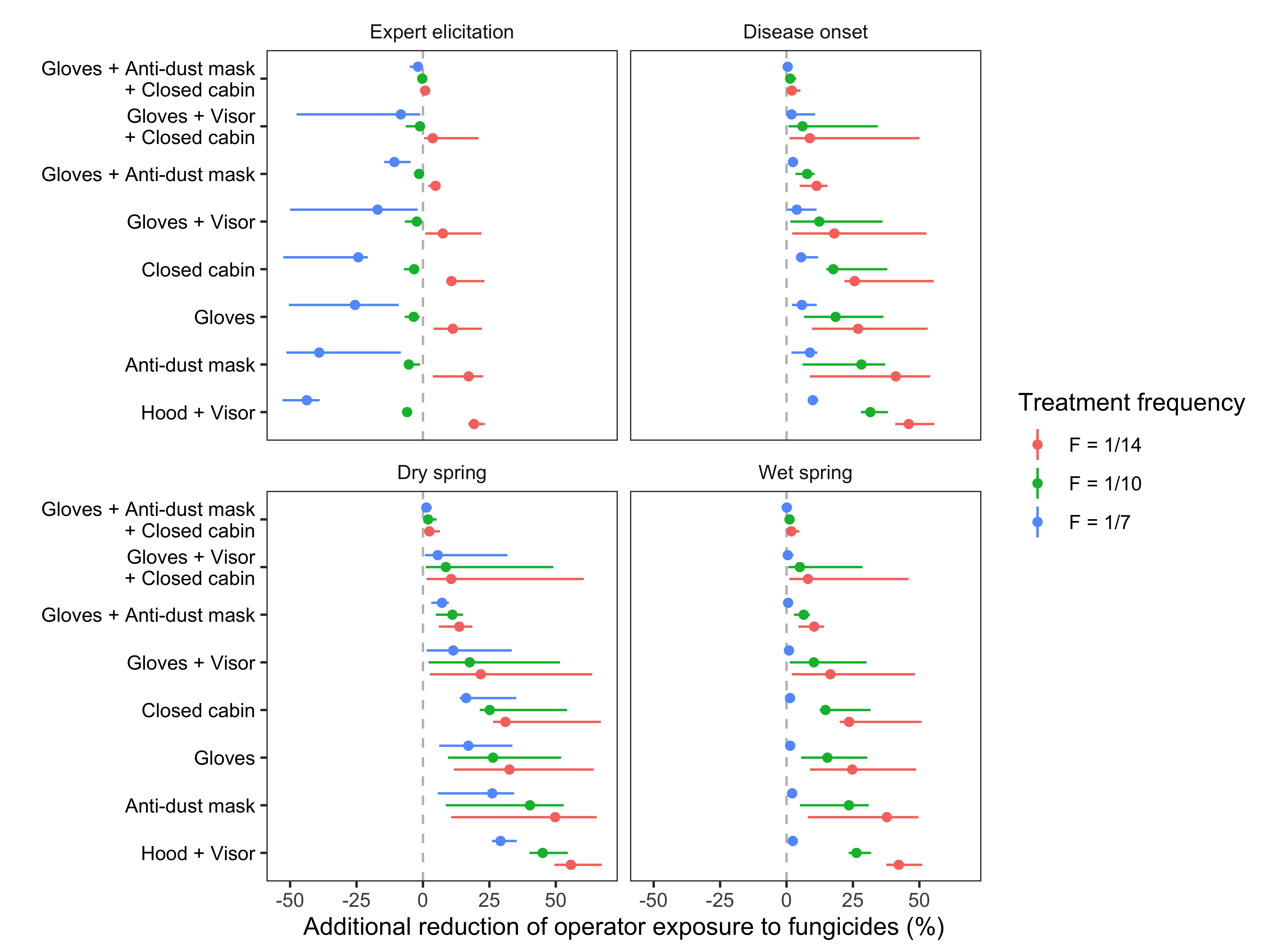


Supplementary Figure 3: Additional reduction of operator exposure resulting from delayed first anti-GDM treatment combined with various operator protection scenarios, according to different GDM control strategies. Each cell represents results for different date of the first application and the color of the boxplots indicates treatment frequency. In each cell, the point represents the median additional exposure reduction across all molecules and horizontal segment represent the range between min and max values.
